# Evaluation of Quality of Life in the Brazilian Graves' Disease Population: Focus on Mild and Moderate Graves' Orbitopathy Patients

**DOI:** 10.3389/fendo.2019.00192

**Published:** 2019-04-04

**Authors:** Danilo Villagelin, João Romaldini, Juliana Andrade, Roberto Santos, Ana Milkos, Patricia Fátima dos Santos Teixeira, Laura S. Ward

**Affiliations:** ^1^Endocrinology and Metabolism, School of Medicine, Pontifical Catholic University of Campinas, Campinas, Brazil; ^2^Laboratory of Cancer Molecular Genetics, School of Medicine Sciences, Campinas State University, Campinas, Brazil; ^3^Hospital do Servidor Público Estadual, São Paulo, Brazil; ^4^Endocrinology, Federal University of Rio de Janeiro, Rio de Janeiro, Brazil

**Keywords:** thyroid, Graves disease, Graves orbitopathy, quality of Life, hyperthyroidism

## Abstract

**Background:** Quality of life (QoL) studies in patients with mild to moderate Graves' orbitopathy (GO) are scarce.

**Methods:** The original GO-QoL questionnaire was translated to Portuguese and administered to 323 patients with Graves' disease. The clinically active score (CAS) was used to evaluate GO activity, and the NO SPECS and EUGOGO classifications were used to estimate GO severity.

**Results:** The internal consistency of the GO-QoL, evaluated using Cronbach's alpha, was optimal. In people with Graves' disease and long-duration GO, both visual function and appearance scores were negatively associated with the CAS and NOSPECS and EUGOGO classifications (*P* < 0.001). Asymmetry and proptosis were significantly associated with the visual function and appearance domains, and diplopia was related to the visual function score. In addition, multivariate regression stepwise analysis revealed that disease severity, according to the EUGOGO classification, was associated with the visual function and appearance scores; asymmetry, presence of proptosis, and young age were associated with the appearance score (*P* < 0.001). The visual function and appearance scores were negatively correlated with the CAS and NOSPECS and EUGOGO classifications (*P* < 0.001).

**Conclusion:** Graves' orbitopathy has a negative impact in QoL in patients with mild to moderate disease, even after an extended period, rendering GO a chronic disease. The GO-QoL questionnaire can be helpful in identifying patients in need of attention and support.

## Introduction

Graves' orbitopathy (GO) is the most common and important extrathyroidal manifestation of Graves' disease (GD) (1, 2). It is present in a mild form in 20% of GD patients, showing minimal eyelid swelling, lid retraction, and proptosis with little or no extraocular muscle dysfunction (3). Moderate forms occur in 5–10% of GD patients and severe forms in 1% of patients (1). Although autoimmune mechanisms are clearly involved, given the role of thyrotropin receptor antibodies and several cytokines, its precise pathogenesis remains poorly understood (1, 4, 5) The onset and progression of GO seem to be influenced by several factors such as smoking, thyroid dysfunction, a strong genetic influence (6, 7), and choice of treatment modality (2). Active GO disease involves some degree of motility dysfunction, diplopia, and sight-threatening conditions such as dysthyroid optic neuropathy and serious corneal exposure, resulting in corneal ulceration, or scarring (2, 4). Visual limitations, changes in appearance, and reduced quality of life (QoL) have been found in severe forms of the orbital disease. However, health-related QoL is usually overlooked in daily clinical practice. The mild and moderate forms can also have significant impacts on psychological functioning in QoL, as described by Wickwar et al. (3).

The concept of QoL is relatively new and has acquired growing importance in recent decades. Certain guidelines reinforce its use in clinical practice because the inclusion of physical, mental, and social aspects, though difficult to measure, reflects a better approach to patient health management (2, 8, 9). Nevertheless, data on QoL measures in GO patients is scarce because most studies evaluate functional health and QoL related to other eye diseases (10–14). Terwee et al. (15) developed a specific QoL questionnaire related to GO, the GO-QoL, for Dutch patients, and it has been used to measure changes in QoL following GD and GO treatments (16, 17). The GO-QoL correlates lower QoL with more severe GO in various populations (18, 19).

The European Group of Graves‘ Orbitopathy (EUGOGO) consortium recommended that a disease-specific QoL, rather than a general questionnaire, be used. Because most studies include patients with severe forms of GO, we evaluated the influences of mild and moderate GO on QoL by using the GO-QoL with a large cohort of Brazilian GO patients. Additionally, we assessed correlations between inflammatory activity, measured using the clinical activity score (CAS), and clinical severity, as determined by the NOSPECS (no physical signs or symptoms, only signs, soft tissue involvement, proptosis, extraocular muscle signs, corneal involvement, sight loss) classification.

## Materials and Methods

### Patients and QoL Questionnaires

The study was carried out in three Brazilian University Hospital centers. After institutional research ethics approval, a total of 323 consecutive GO patients with mild to moderate forms of the disease were eligible (Table 1). The original 16-item GO-QoL questionnaire was divided into visual function and appearance sections (15), excluding the first question about riding a bike because most of our patients (>85%) did not ride a bike. Hence, the visual function section contained seven items. Before administering the GO-QoL, the EUGOGO classification was completed, the CAS was determined, and the NOSPECS classification was assigned. Additionally, a routine clinical examination and laboratory tests were performed. The duration of GO was estimated using onset data (first signs and/or symptoms). The CAS was used to evaluate the inflammatory activities of GO according to Mourits et al. (20). A total score ranging from 0 to 7 was reached by assigning one point for each GO characteristic: spontaneous retrobulbar pain, pain on attempting an upward or downward gaze, eyelid redness, conjunctival redness, eyelid swelling, inflammation of the caruncle, and conjunctival edema. The severity of GO was estimated using the NOSPECS classification: (21) Class 0 is assigned to patients with no physical signs or symptoms; Class I, signs only (e.g., eyelid retraction); Class II, soft tissue involvement; Class III, proptosis; Class IV, significant extraocular muscle signs; Class V, corneal involvement; and Class VI, sight loss (e.g., optic nerve compression). The EUGOGO classification (2) divides patients into three groups. The first comprises patients with mild GO, usually displaying one or more signs such as minor lid retraction (<2 mm), mild soft-tissue involvement, exophthalmos (<3 mm above normal for race), intermittent diplopia, and corneal exposure responsive to lubricants. The second comprises those with moderate to severe GO or GO that is not sight-threatening. The eye disease usually displays two or more of the following: lid retraction (≥2 mm), moderate or severe soft-tissue involvement, exophthalmos (≥3 mm above normal for race), and non-constant or constant diplopia. The third comprises those with sight-threatening and dysthyroid optic neuropathy or corneal breakdown. A scale rule was used to measure eyelid width, and a Hertel exophthalmometer was used to measure proptosis. Intermittent and permanent diplopia was also evaluated. Asymmetry was defined as a difference of at least 3 mm between the eyes. Each medical center had an experienced doctor trained to administer the questionnaires. Exclusion criteria were ophthalmic diseases such as cataract, age-related macular degeneration, and glaucoma that could influence answers to the QoL questionnaire. To avoid confounders, clinical, and laboratory euthyroid patients were the only people included in this study.

**Table 1:** Baseline characterisitcs of the study GO patients.

	**Parameters**	**Values**
Patients, *n*		323
Age, year		48.97 ± 14.77
Sex, male/female		56/267
Smokers, (positive)%		22.22
Duration of GO, year		7.12 ± 6.66
Diplopia, (positive)%		3.15
Asymmetry, (positive) %		18.63
**PROPTOSIS, mm**		
	Right eye	17.95 ± 3.71
	Left eye	17.94 ± 3.6
**LID FISSURE WIDTH, mm**		
	Right eye	9.56 ± 1.95
	Left eye	9.64 ± 1.98
**CAS SCORE**, ***n***		
	0–1	246
	2–5	77
**NO SPECS SCORE**, ***n***		
	0–1	204
	2–4	119
**EUGOGO**, ***n***		
	Mild	229
	Moderate to Severe	94
Serum TSH (mU/L)		2.39 ± 1.27
Serum Ft4 (ng/dL)		1.21 ± 0.33
Serum TRAb (positive) %		91.8
Antithyroid drug therapy, *n*		248
Radioiodine therapy, *n*		70
Thyroidectomy, *n*		5

*GO, Graves' orbitopathy; TRAb; CAS, clinical activity score; NO SPECS, EUGOGO; values are given as number (n), percentages (%), and mean ± standard deviation*.

### Laboratory Analysis

Serum-free thyroxine and thyroid stimulating hormone levels were determined using chemiluminescence assays (DPC Immulite system). Thyrotropin receptor antibody levels were determined using electrochemiluminescence assays (Elecsys 2010, Roche Diagnostics).

### Statistical Analysis

Samples were separated according to the variables studied. Frequency tables were constructed for categorical variables and were described using *n* and percent. Numerical variables were described using the mean, standard deviation, and median. The chi-square test was used to compare categorical variables between classifications and, when necessary, the Fisher exact test was used. The Mann-Whitney and Kruskal-Wallis tests were used with numerical variables. Correlations between the CAS, EUGOGO classification, NOSPECS classification, and the GO-QoL score were determined using the Spearman rank correlation. Univariate and multivariable logistic regression were performed to investigate the influences of age, sex, smoking, diplopia, asymmetry, proptosis, CAS, NO SPECS, EUGOGO classification, and duration of GO reflected in the GO-QoL score. Cronbach's alpha was used to evaluate internal consistency based on correlations between items within a subscale of the QoL questionnaire.

Statistical significance was recognized when *P* < 0.05. All analyses were performed using SAS (Statistical Analysis System) and BioStat Pro 6.1.7.

## Results

**Figure 1:**
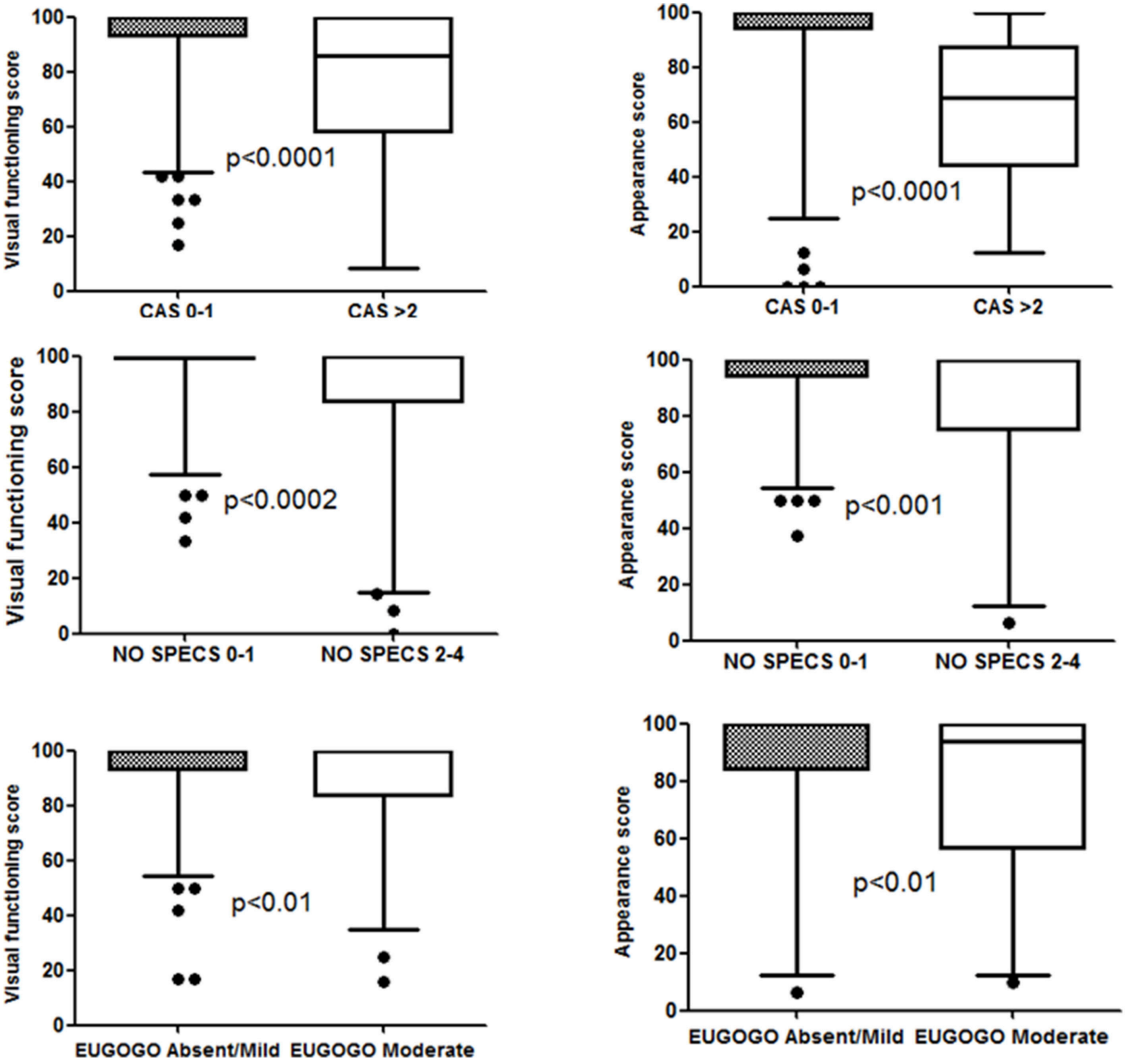
The effect of clinical activity scores (CAS), NO SPECS scores, and EUGOGO scores on Graves' ophthalmopathy quality of life (GO-QoL) scores of functional visual and appearance domains. The Mann–Whitney test was used for differences between the two groups. The box encloses the interquartile range, the median is marked by a line within the box, and the “whiskers” extend from the interquartile range to the 2.5 and 97.5 percentiles.

Table 1 shows the baseline characteristics of the sample. Of the 323 included GO patients with mild to moderate forms of the disease, 267 (83.43%) were women. Mean (±standard deviation) age was 48.97 ± 14.7 years. The duration of GO averaged 7.12 ± 6.66 years. All patients had clinical and laboratory characteristics of euthyroidism. The internal consistency of GO-QoL was found to be optimal and well-preserved in both domains. Cronbach's alpha was 0.8425 for functional visual domains and 0.9163 for appearance domains. The sample was divided in two groups. Members of one had absent or mild GO, CAS of 0 or 1 (*n* = 246), NOSPECS classifications of 0 or 1 (*n* = 204), and mild GO according to EUGOGO classifications (*n* = 229). Members of the other had moderate GO, CAS of 2 to 5 (*n* = 77), NOSPECS classifications of 2 to 4 (*n* = 119), and moderate to severe GO according to EUGOGO classifications (*n* = 94). About three percent of the sample had diplopia at the time of QoL testing. However, a large majority of patients with diplopia had CAS exceeding 2, NOSPECS classifications of 2 to 4, and were classified as moderate by their EUGOGO classifications (Table 2). Additionally, as Figure 1 shows, GO patients with CAS exceeding 2, NOSPECS classifications of 2–4, and EUGOGO classifications indicating moderate eye disease had significantly lower scores in both visual functioning and appearance domains. Table 3 shows that in the univariate logistic analyses, CAS, NOSPECS classification, EUGOGO classification, asymmetry, and proptosis were variables associated with visual function and appearance domains, but diplopia was associated with only the visual function domain. In the multivariate analysis using stepwise regression, only the EUGOGO classification was associated with both the visual functioning and appearance domains. Furthermore, asymmetry, proptosis, and young age were associated with only the appearance domain. Finally, as Table 4 shows, Spearman's rank correlation analysis indicates a negative correlation between the visual function and appearance domains and the three GO classifications (*P* < 0.001).

**Table 2:** Characteristics of the selected GO patients from the inicial study: comparison of clinical variables among the GO classifications.

	**CAS**			**NO SPECS**			**EUGOGO**		
	**0–1**	**2–5**	***p*-value**	**0–1**	**2–4**	***p*-value**	**mild**	**moderate a severe**	***p*-value**
Patients, n	246	77	-	204	119	-	229	94	-
Age, years	48.99 ± 15.31	48.92 ± 12.99	0.995	49.53 ± 15.41	48.02 ± 13.62	0.385	49.09 ± 15.25	48.68 ± 13.59	0.972
Duration of GO, year	7.37 ± 6.88	6.32 ± 5.87	0.228	7.58 ± 7.10	6.34 ± 5.77	0.106	7.32 ± 6.94	6.63 ± 5.91	0.398
Sex, male/female	38/208	18/59	0.121	32/172	24/95	0.360	36/193	20/74	0.258
Diplopia(positive) %	1.22	9.85	<0.002	0.49	7.89	<0.001	0.44	10.11	<0.0001
Asymmetry (positive) %	15.19	30.43	<0.02	12.93	26.14	<0.02	12.06	33.33	<0.0003
Proptosis (positive) %	22.15	71.74	<0.0001	12.07	61.36	<0.0001	13.48	77.78	<0.0001
Serum TSH(mU/L)	2.45 ± 1.29	2.58 ± 1.34	0.445	2.40 ± 1.25	2.55 ± 1.36	0.214	2.39 ± 1.29	2.60 ± 1.30	0.186
Serum FT4(pmol/L)	1.17 ± 1.29	1.20 ± 0.26	0.426	1.18 ± 0.32	1.23 ± 0.31	0.171	1.19 ± 0.31	1.30 ± 0.37	0.098
Antithyroid drugs therapy, n	189	59	0.724	157	91	0.291	176	72	0.531
Radioiodine therapy, n	53	17	0.531	44	26	0.871	49	21	0.184
Thyroidectomy, n	4	1	0.823	3	2	0.531	4	1	0.872

*Values are number of patients (n), percentages or mean ± standard deviation*.

**Table 3:** Associations between QOL score and clinical variables, CAS, NOSPECS, and EUGOGO domains by using univariate and multivariate regression stepwise analysis.

	**Functional visual score**	**Appearance score**
	***p*-value**	***p*-value**
**UNIVARIATE ANALYSIS**		
Age	0.509	0.032
Sex	0.627	0.965
Duration of GO	0.284	0.859
Diplopia	<0.05	0.445
Asymmetry	<0.05	<0.0001
Proptosis	<0.0001	<0.0001
CAS	<0.0001	<0.0001
NO SPECS	<0.0001	<0.0001
EUGOGO	<0.0001	<0.0001
**MULTIVARIATE ANALYSIS**		
Age		0.007
Asymmetry		<0.0001
Proptosis		<0.0001
EUGOGO	<0.0001	<0.0001

**Table 4:** Correlation coefficients between QoL domains and questionnaire scores of CAS, NOSPECS, and EUGOGO classifications in go patients.

	**CAS**	**NOSPECS**	**EUGOGO**
Functional visual	*r*_s_ = −0.548; *p* < 0.0001	*r*_s_ = −0.529; *p* < 0.0001	*r*_s_ = −0.544; *p* < 0.0001
Appearance	*r*_s_ = −0.626; *p* < 0.0001	*r*_s_ = −0.646; *p* < 0.0001	*r*_s_ = −0.600; *p* < 0.0001

*Spearman's rank correlation coefficients are shown*.

## Discussion

The concept of QoL includes physical, mental, and social aspects (1, 8). Thus, it is easy to understand that patients with severe GO, particularly those with sight impairment, have low QoL scores. However, most patients in clinical practice present with mild to moderate forms of GO (2, 4). In chronic diseases, the general health-related QoL questionnaire includes several short and simple self-assessment questions and measures the most important aspects of QoL (11). Studies using general questionnaires with GO patients demonstrate that they are disturbed and dissatisfied with their appearance (10). Likewise, a Swedish study using patients with hyperthyroidism and GO treated with radioiodine or with antithyroid drugs showed similar results when the MOS-SF36 was used. However, patients who developed or had worsening GO had decreased QoL independent of GD treatment (22). A specific vision test such as The National Eye Institute Visual Function Questionnaire was instituted to evaluate the impact of chronic eye disease. Patients with mild to severe GO indicated worse QoL, particularly when diplopia was present (23). Terwee et al. developed the first questionnaire specific to patients with GO (the GO-QoL), adding information to traditional physiological and biological measures of health status (15, 24) and correlating with patients who received orbital surgery (16). The TED-QoL instrument is a less complex questionnaire than the GO-QoL instrument in that it has three instead of 16 items; however it is used infrequently (25, 26). Our findings using the GO-QoL questionnaire agree with those obtained in other countries (27, 28). We also confirmed data reported by others: that the GO-QoL questionnaire is associated with the CAS and the NOSPECS and EUGOGO classifications (27, 29). Furthermore, this study is one of the first to show that patients with mild to moderate forms of GO have very low QoL scores even after lengthy follow-up (7 years), suggesting they should receive special attention and psychological support. Even with the size of our cohort and the strength of our results, the number of patients with diplopia was small, possibly because diplopia usually improves with treatment. However, the presence of diplopia as well as asymmetry and proptosis are factors that significantly impair QoL. No matter the classification, visual functioning, and appearance domains yielded lower scores when CAS exceeded 2, the NOSPECS classification was 2 to 4, and the EUGOGO classification was moderate. Thus, greater changes in the eye resulted in poorer QoL. These results highlight that EUGOGO and GO-QoL classifications are efficient tools for evaluating GO patients. However, this work has limitations including a lack of controls and QoL evaluations prior to GD treatment. In summary, as with other studies (19, 27–30), both EUGOGO and GO-QoL are useful tools for assessing QoL in GO patients with mild to moderate forms of the disease. Furthermore, GO decreases QoL, and its effects remain even after a lengthy follow-up, suggesting that GO behaves as a chronic disease. An important corollary from these data is that changes in vision and appearance lead to difficulties in daily visual functioning and a declining psychological state. With the aid of the GO-QoL questionnaire, patients requiring specific support might be identified.

## Data Availability

All datasets generated for this study are included in the manuscript and/or the supplementary files.

## Ethics Statement

This study was carried out in accordance with the recommendations of Ethics Committee, with written informed consent from all subjects. All subjects gave written informed consent in accordance with the Declaration of Helsinki. The protocol was approved by the Instituto de Assistência Médica ao Servidor Público Estadual—IAMSPE Ethics Committee.

## Author Contributions

DV, JR, and JA prepared the manuscript and analyzed the data. JA and RS created the charts and figures. RS, AM, and PT managed and verified the collected data. DV, AM, and PS were responsible for interview the patients. LW and JR provided overall guidance and edited the manuscript. The final manuscript was read and approved by all authors.

### Conflict of Interest Statement

The authors declare that the research was conducted in the absence of any commercial or financial relationships that could be construed as a potential conflict of interest.
